# Wearable GPS and Accelerometer Technologies for Monitoring Mobility and Physical Activity in Neurodegenerative Disorders: A Systematic Review

**DOI:** 10.3390/s21248261

**Published:** 2021-12-10

**Authors:** Mícheál Ó. Breasail, Bijetri Biswas, Matthew D. Smith, Md Khadimul A. Mazhar, Emma Tenison, Anisha Cullen, Fiona E. Lithander, Anne Roudaut, Emily J. Henderson

**Affiliations:** 1 Population Health Sciences, Bristol Medical School, University of Bristol, 1-5 Whiteladies Road, Bristol BS8 1NU, UK; micheal.obreasail@bristol.ac.uk (M.Ó.B.); matthew.smith@bristol.ac.uk (M.D.S.); qc20383@alumni.bristol.ac.uk (M.K.A.M.); emma.tenison@bristol.ac.uk (E.T.); hk21935@bristol.ac.uk (A.C.); Fiona.lithander@bristol.ac.uk (F.E.L.); emily.henderson@bristol.ac.uk (E.J.H.); 2 Department of Electronic and Electrical Engineering, Computer Science and Mathematics, University of Bristol, Bristol BS8 1TH, UK; 3 Older Peoples Unit, Royal United Hospital NHS Foundation Trust, Bath BN1 3NG, UK; 4 Department of Computer Science, University of Bristol, Bristol BS8 1TH, UK; Anne.Roudaut@bristol.ac.uk

**Keywords:** Parkinson’s disease, Alzheimer’s disease, remote monitoring, sensors, GPS, accelerometry, movement/mobility, physical activity, wearable technology

## Abstract

Neurodegenerative disorders (NDDs) constitute an increasing global burden and can significantly impair an individual’s mobility, physical activity (PA), and independence. Remote monitoring has been difficult without relying on diaries/questionnaires which are more challenging for people with dementia to complete. Wearable global positioning system (GPS) sensors and accelerometers present a cost-effective and noninvasive way to passively monitor mobility and PA. In addition, changes in sensor-derived outcomes (such as walking behaviour, sedentary, and active activity) may serve as potential biomarkers of disease onset, progression, and response to treatment. We performed a systematic search across four databases to identify papers published within the past 5 years, in which wearable GPS or accelerometers were used to monitor mobility or PA in patients with common NDDs (Parkinson’s disease, Alzheimer’s disease, motor neuron diseases/amyotrophic lateral sclerosis, vascular parkinsonism, and vascular dementia). Disease and technology-specific vocabulary were searched singly, and then in combination, identifying 4985 papers. Following deduplication, we screened 3115 papers and retained 28 studies following a full text review. One study used wearable GPS and accelerometers, while 27 studies used solely accelerometers in NDDs. GPS-derived measures had been validated against current gold standard measures in one Parkinson’s cohort, suggesting that the technology may be applicable to other NDDs. In contrast, accelerometers are widely utilised in NDDs and have been operationalised in well-designed clinical trials.

## 1. Introduction

### 1.1. Neurodegenerative Disorders

Neurodegenerative disorders (NDDs) constitute an increasing global burden. Alzheimer’s disease (AD) is the most common neurodegenerative disorder. Dementia has a high prevalence affecting 50 million people worldwide and approximately 850,000 people in the UK alone [1]. Parkinson’s disease (PD) is the second most common disorder which can have profound effects on both mobility and cognition with other neurological disorders such as vascular dementia and the collection of motor neuron diseases also contributing to the significant number of cases and burden of disease.

NDDs are characterised by a progressive loss of neural cells over time [2]. This can be caused by a variety of processes, ranging from the accumulation of amyloid plaques leading to neuronal death in AD [2] to the aggregation of intracellular alpha-synuclein that characterises PD [3]. The underlying cause of these processes are still enigmatic and disease-modifying treatments have not yet been established in routine clinical practice. Higher order functions such as walking, memory, and language most frequently occur as the symptoms of NDDs [4]. The phenotype of symptoms depends on the underlying pathology; AD manifests as a gradual loss of memory evolving over time into a more global brain dysfunction, whilst PD is characterised by progressive difficulty with movement (in particular walking) and memory, and cognitive symptoms usually emerge and worsening as the disease progresses [5]. As well as causing distressing symptoms for both an individual and their family, a significant socioeconomic cost is associated with the spectrum of neurodegenerative conditions. In Europe, the combined economic impact of dementia and PD has been estimated to be nearly 120 billion euro, with almost half attributed to loss of productivity [6]. Therefore, the importance of maintaining people’s function in society is self-evident.

### 1.2. Mobility and Physical Activity as an Invaluable Predictor for NDD

Mobility concerns the ability of an individual to move within their environment and impaired mobility negatively impacts a person’s quality of life as well as their participation within society [5]. Problems with movement are implicit in PD, with symptoms such as stiffness, lack of balance, and a tendency to fall leading to decreased confidence mobilising [7]. Mobility can also be affected in AD, with a propensity to fall as well as cognitive dysfunction precipitating difficulties undertaking day-to-day activities. This impacts functionally, attenuates enjoyment of hobbies and activities, and ultimately can lead to safety implications associated with leaving the home unsupervised [8]. The amount of time spent outside and movement within the home represents a marker of health with associated impact on quality of life [9]. Quantification of mobility and physical activity (PA) in neurodegenerative diseases, including with disease progression, offers the opportunity to target these metrics with specific interventions that may feasibly maintain, or improve quality of life and well-being [9]. Thus, the extent of an individual’s ability to travel within the world may represent a measurable biomarker, important for monitoring the effect of interventions including emergent and novel disease modifying therapies [10].

### 1.3. Current Solution to the Mobility Problem

Objectively measuring an individual’s location and mobility has become increasingly possible due to the widespread integration of GPS and similar technologies (e.g., Galileo and GLONASS) in consumer devices such as smartphones, navigation systems, and increasingly in wearables (e.g., GPS watches). These devices can accurately and precisely determine position at any time point to within a matter of metres. Data obtained from GPS devices can be used as input data to generate mobility indicators that describe an individual’s daily mobility patterns [11]. Fillekes et al. recently described a conceptual framework for the use of GPS in health and ageing research and defined space, time, and scope of movement as the primary categories into which mobility indicators could be grouped [12]. 

Despite the increased use of wearable GPS in ageing research, most studies have reported a limited set of mobility indicators [11]. The research suggests that GPS-based technologies may be capable of differentiating mild-to-moderate AD patients from people without AD [13]. In validation studies, GPS-based technologies that measures location and mobility have been found to show reasonable agreement with the current gold standard methods of an interview and a diary for determining life space mobility [12,14,15,16]. GPS-based technologies hold the advantage of being able to passively monitor the mobility of patients with minimal burden and minimise recall bias, if the device is worn [12]. This simplicity of wearable solutions can be of considerable use in the context of neurodegenerative disease where cognitive function and memory may be diminished.

Wearable accelerometers or inertial measurement units (IMU) have been widely used in medical research [17,18], in particular, for conditions where movement may be affected. Basic accelerometers measure acceleration against a reference and can provide simple ratios of time spent in active or sedentary modes. However, with appropriate processing, the data can be classified by the intensity of the activity (i.e., light or moderate exercise) or estimate distance travelled (i.e., steps). Wearable accelerometers have been used in two principal contexts: the first is the use of multiple IMUs to examine specific abnormal parameters of movement (i.e., gait speed and stride length, or incidence of freezing of gait in PD) [19,20] and the second includes studies where overall PA and behaviour patterns of specific patient populations have been described (i.e., distance and time spent moving), with and without comparison to healthy controls [19,20].

### 1.4. Processing Tools and Algorithms Used for Data Analysis

When collecting data at high frequency and over long periods of time, wearable GPS and accelerometers produce large volumes of raw data. The processing of these data is possible through standard statistical software, although this may be slow. Access to a satisfactory graphics processing unit (GPU) in a high-performance computing hardware environment coupled with machine learning (ML) or deep learning networks offers an alternative. ML is a form of artificial intelligence (AI) which enables software applications to identify and accurately predict patterns through learning from examples and analogy. Some of the commonly used methods include neural network, convolution neural network, recurrently neural network, K-means clustering, and support vector analysis (SVM). A challenge associated with these computational methods is that they are specific to detecting a certain feature. Due to the complexity and overlapping symptoms of NDD, it is difficult to diagnose, monitor, and provide treatment for patients [21]. A deep learning method with feature extraction in an unsupervised manner can improve the accuracy of classification [22]. There are several off-the-shelf devices available and the processing method to analyse the data varies. To address the heterogeneity, sampling rate, and sensor biases of the devices, future research should investigate cross-domain transfer as well as ML and deep learning networks to accommodate this heterogeneity [23].

### 1.5. Contribution of This Paper to the Literature

Despite the increasing ease and ability to examine the mobility of subjects with sensor devices, there has been limited research that has included these technologies in large clinical trials of interventions for neurodegenerative conditions. This is a critical step in the evolution of these technologies, in which the relationship between mobility metrics and specific clinical endpoints can be determined, and therefore allow for their integration into healthcare and rehabilitation [24].

This systematic review aims to consider the literature concerning the use of GPS and accelerometer devices in assessing mobility and PA in individuals with neurodegenerative conditions. Our objectives are:

1. To identify to what extent GPS and accelerometer-derived measures have been used as biomarkers of mobility and PA in clinical research related to neurogenerative disease;
2. To describe the outcome measures reported from these patient populations;
3. To identify studies that have established a relationship between neurodegenerative disease and sensor-derived mobility and PA data.

## 2. Materials and Methods

### 2.1. Search Strategy

We conducted a systematic search of the English language literature, up to 10 September 2021 using OVID with the following four databases enabled: MEDLINE, EMBASE, AMED (Allied and Complementary Medicine), and APA PsycInfo. We restricted the search to human studies conducted within the last five years. Our search was performed as defined by the latest Preferred Reporting Items for Systematic Review and Meta-Analysis (PRISMA) guidelines [25]. The search relied on a combination of disease and technology specific MeSH terms and keywords which were initially searched in isolation and subsequently combined. Examples included: “neurodegenerative disease”, “Parkinsons”, “Alzheimers”, “Global Positioning System”, and “Accelerometry” (Table S1). PICO (population, intervention, comparison, outcome) principles were used for the selection criteria: (P) people with neurodegenerative disease at baseline; (I) wearable devices containing GPS and/or accelerometers; (C) none; (O) mobility measures from wearable technologies.

### 2.2. Eligibility

The search criteria were structured to include studies that encompassed the following: (a) considered groups with five common neurodegenerative disorders (Parkinson’s disease, Alzheimer’s disease, motor neuron diseases/amyotrophic lateral sclerosis, vascular parkinsonism, and vascular dementia); (b) assessed mobility or PA measures derived from GPS or accelerometer sensors; (c) used devices that were “wearable” as they are noninvasive, unobtrusive, and portable tools that can continuously monitor daily PA and mobility [14]. The exclusion criteria included studies (a) where only a smartphone was used; (b) where only step counts were reported; (c) where sensors were used only to validate specific outcomes (i.e., Timed Up and Go), focused on a specific limb movement such as tremor rather than mobility, or reported quantitative outcomes of specific gait parameters (i.e., double-support time, stride length); (d) that did not contain primary data; or (e) did not undergo peer review.

### 2.3. Study Selection

**Figure 1:**
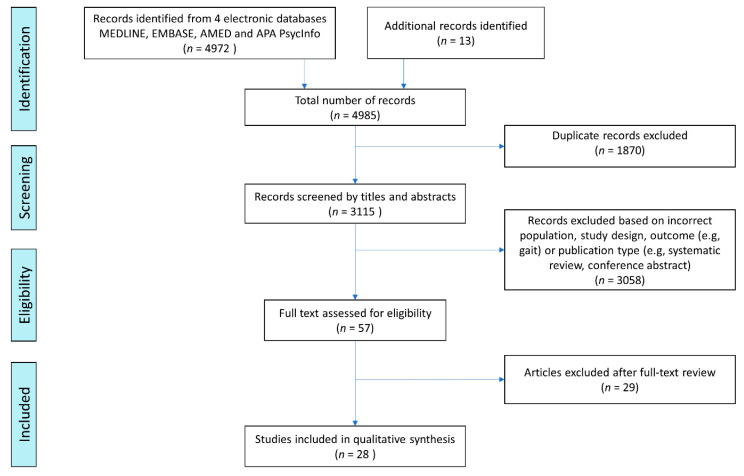
PRISMA flowchart summarising the search process.

All references were uploaded to EndNote reference management software, where duplicates were removed prior to importing to Rayyan for review. The title and abstract reviews were conducted by B.B. in tandem with A.C. and M.D.S. If there was a disagreement on eligibility, M.Ó.B. determined the final decision. Thirteen further papers were identified from the reference sections of a number of relevant review papers and identified texts. Following the screening of titles and abstracts against the eligibility criteria, 3058 papers were excluded, leaving 57 papers for full-text review; 345 papers were identified as conference abstracts and excluded. Others were excluded on the basis of study outcome (e.g., gait) and publication type (e.g., conference abstract or systematic review). A total of 28 papers met the eligibility criteria following the full-text review for data extraction. A summary of our screening process is displayed in Figure 1.

### 2.4. Data Extraction and Study Synthesis

Initial data extraction was conducted by B.B. who reviewed full texts of the identified studies to extract the following: author (year), study population, device site, duration, study design, reported sensor-derived outcome measures, and key findings. It was then cross-checked by F.E.L. for accuracy. The PRISMA diagram shows the literature identification and screening process (Figure 1). Heterogeneity precluded quality assessment.

## 3. Results

### 3.1. Study Characteristics

The total number of papers found was 4985, with 3115 papers remaining after deduplication. Figure 1 summarises the search process and Table 1 describes the 28 studies that were included in the analyses. In the identified literature, PD was the most widely researched NDD (17 studies), followed by dementias (10 studies), and MND/ALS (1 study). A single study was found to have used both wearable GPS and accelerometry in PD, while a further 27 studies used an accelerometry across a number of NDDs (Table 1). Devices were worn at a number of anatomical sites although the wrist and hip were the most routinely used sites (Table 1). Most studies were conducted in high-income countries with the exception of one Chinese study; no studies from low- and middle-income countries were identified.

### 3.2. Use of Wearable GPS to Measure Mobility in NNDs

Zhu et al. used a WIMU-GPS (Realtrack Systems S.L., Almería, Spain) unit, which combined accelerometry and GPS, in 54 Canadian older adults with early- to mid-stage PD, for 14 days [15]. The study compared GPS-derived “hourly frequency” defined as the number of trips outside of the home per hour sampled, “daily duration” defined as percentage of total time sampled per day that participants were outside of home, and “life space size” to self-reported measures of the same variables [15]. The authors reported moderate agreement between paper-based and digital measures of mobility and life space, noting the floor and ceiling effects of the standard LSA measure of life space, which were overcome when GPS was used. Participant attrition in the study was high at ~40%, but this was in keeping with similar studies in other populations [12,63,64]. Attrition was attributed to a number of factors including forgetting to charge the battery, not continuously wearing the device, and equipment errors with 7 days’ worth of data loss occurring in 6 participants.

### 3.3. Use of Accelerometry to Measure PA in NDDs

Accelerometers are widely used in NDDs to measure PA, although studies have also reported accelerometer-derived variables related to gait and/or sleep (Table 1). Most studies used a single dedicated device per patient, three studies placed multiple units on different anatomical sites or compared different models of device within the same patient(s) [20,43,59]. The reported accelerometer-derived measures of PA varied greatly among studies, which in part is explained by the different technologies used and their varying anatomical positioning. Most studies, regardless of model of device used, were able to give a measure of step count (19/28) or time spent in active or sedentary states (19/28). Four of the 28 studies also reported sleep-related measures such as sleep duration and sleep activity, in addition to PA.

Wrist and hip worn sensors such as the Actigraph (ActiGraph LLC, Pensacola, FL, USA) were used to provide more detailed measures of physical activity, such as intensity levels as compared with those fitted to the lower back such as the DynaPort Minimod (McRoberts BV, The Hague, The Netherlands) which were mostly used to define the amount of time spent in different postures including lying, sitting, standing, and walking [26]. Eleven studies explored associations between objective accelerometer-derived measures and widely used in-person assessments (e.g., TUG) and paper-based tools that assess function (e.g., ALS Functional Rating Scale-Revised), mobility (e.g., Parkinson’s Disease Questionnaire (PDQ) mobility scores), or PA (e.g., Physical Activity Scale for the Elderly) [20,26,27,28,34,36,39,43,46,59]. Furthermore, nine of the studies explored relationships between sensor-derived activity measures and disease stage, duration, or progression [20,27,28,30,34,35,39,40,45]. One study proposed that assessing physical behaviour patterns, particularly sedentary bouts, could potentially be used to discriminate between PD with mild cognitive impairment and PD with dementia [40]. Kim et al. reported that, in PD, the number of steps recorded by wrist-worn devices were significantly higher than those recorded by waist-worn devices (*p* < 0.001). Increased tremor and dyskinesia explained 19% of the variation in the difference of daily average steps between these two sites [20].

## 4. Discussion

In this section, initially, we discuss the overall findings of the review followed by a broader discussion in the context of the wider use of GPS and accelerometers in NDDs. Finally, we consider future research that this work opens up and potential limitations of this.

### 4.1. Summary of Overall Findings

In this review, we have systematically identified primary research papers that have measured mobility and PA with wearable digital devices in individuals living with prevalent and burdensome neurological disorders. The eligibility criteria were selected to focus our review on NDDs. Although these are caused by differing pathological processes, all share a common trajectory of progressive neuronal loss correlating with decline in function. The selected conditions were based on their high population incidence, and therefore higher likelihood of detecting studies in the literature. Nevertheless, a number of studies on Huntingdon’s disease (HD) were found (not included in our search criteria), despite this being less common than ALS. This is likely due to the longer duration of illness in a relatively young age group for HD, frequently diagnosed at the pre-symptomatic stage, as opposed to the rapid and disabling course seen in ALS. We focused specifically on wearable GPS devices, as they present a novel and robust method to capture continuous noninvasive geolocation and spatial mobility data from patients with NDDs such as dementia for whom carrying a non-worn device may be problematic due to cognitive impairment. For accuracy, the vast majority of accelerometers designed to measure PA are wearable, worn or attached to the body at specific anatomical sites (i.e., wrist), or incorporated in a specific housing such as a belt close to the body. The 28 identified papers demonstrated the predominance of accelerometers (*n* = 28) being utilised more than wearable GPS (*n* = 1). These technologies have been most widely studied in PD with all but one study having been performed in high income countries. The majority of studies were cross-sectional in nature, with sampling periods ranging from 24 h to 3 months. Seven days was the most common measurement period and studies typically excluded data from days with less than 10 h of recording time.

### 4.2. Current Usage of GPS and Accelerometers and Their Potential

Fundamentally, GPS and accelerometry measure different domains but when combined measures derived from these sensors can help to build a holistic view of life space and behaviour patterns. Zhu et al. used integrated accelerometry and GPS (WIMU-GPS) to quantify trips, duration of time outside the home and life space, and the spatial area through which an individual moves [15]. Positive signals indicate digital methods might have surpassed diary-based measures for capturing mobility data and that GPS-based quantification of life space mobility may be a truer reflection of mobility than the gold standard LSA due to floor and ceiling effects. While accelerometer data were not reported, several examples of the quantification of PA in PD and in other NDDs were found in the literature. This suggests that the use of accelerometry in NDDs has the potential to provide continuous remote monitoring of patient PA, behaviour patterns, and sleep activity. Additionally, in complex diseases, the use of accelerometers may present an objective method for monitoring disease progression or response to therapy. The possibility to conduct remote assessments validated by accelerometry such as the Timed Up and Go (TUG) may also allow clinicians to make decisions about treatment without necessarily seeing the patient in a clinic. However, a number of important caveats need to be considered when deciding how best to apply these techniques in patients, as data obtained from accelerometers can vary based on the chosen anatomical site (i.e., wrist versus hip) or disease-specific symptoms may influence measures at one anatomical location but not others. This was apparent from the variability reported by Kim et al., i.e., hyperkinesia associated with tremor and/or dyskinesia can interfere with step detection [20].

### 4.3. Strengths, Limitations, and Acceptability of Wearable GPS

At present, there are several barriers to the widespread incorporation of these technologies in clinical research. As noted in this review, wearable GPS-based technologies have been used much less. often than accelerometers containing activity trackers which have been used widely in NDDs. Currently, a major limitation of wearable GPS-based technologies is that battery life between recharging cycles is typically in the range of 10–12 h when in active use. Although it is possible to prolong battery life by lowering the frequency of measurement, this comes at the expense of accuracy. For this reason, studies that have used wearable GPS devices pragmatically instructed patients to wear the devices during waking hours only [15] or when leaving the home. While such approaches are acceptable given the low accuracy of GPS indoors, relying on patients to remember to put/power the device back on may not be practical in patients with dementia. Although outside the scope of this review, several methodologically rigorous studies which used non-wearable-GPS devices with longer battery lives were identified during the study selection [65,66,67,68]. The acceptability of GPS devices to patients may also present a barrier to use, aspects such as the size, shape, and materials used, in addition to the location on the body where it is worn and the robustness given the prevalence of symptoms of, for example, agitation in dementia and dyskinesia in Parkinson’s disease. One study from our search reported on device acceptability and found that armbands containing accelerometers were not well tolerated in dementia patients, with a majority failing to meet the study’s defined valid wear-time criteria of ≥21 h [51].

Megges et al. investigated the acceptability of two different models of GPS watches in both patients with dementia and their caregivers, with 17 patient–caregiver dyads rating device aspects such as usability, telephone function, overall design features, font, buttons, and battery [69]. The results of the International Standardization Organisation Norm (ISONORM) 9241/10 usability scale for both products ranged from fair to good, but product satisfaction with both products was significantly lower at home [69]. Some research has also been undertaken to identify barriers to the adoption of GPS technologies for dementia care through interdisciplinary stakeholder meetings [70]. 

### 4.4. Strength, Limitation and Acceptability of Wearable Accelerometers

Accelerometer-based devices have the advantage of consuming less power, facilitating continuous wear for days and weeks; however, with current technology no contextual information is usually obtained on behaviour type without corresponding video or meticulous observer documentation. Transparency can be an issue, and reporting can vary greatly between studies, particularly, when proprietary algorithms are used and may change without the end-user’s knowledge, which can present particular difficulty for longitudinal data collection. Data processing practices also vary greatly and make it difficult to compare studies and patient populations. Moreover, policies for classifying zero count data as non-wear can lead to large differences in the estimation of PA and sedentary time. We note that of those papers detailing algorithm use, many simply reported using the manufacturer’s proprietary algorithms to process and analyse their raw data. The use of specific algorithms, other than predefined cuff-offs or manufacturer algorithms, to determine non-wear time [32,71] were reported in two studies [31,39]. A number of studies [28,34,36] reported applying previously published algorithms for wrist-worn accelerometry in older adults [37] or PD-specific algorithms [29,38]. Elazari et al. used support vector machine (SVM) to discriminate features between PD and healthy older adults [42]. Buckley et al. quantified walking in relation to volume, pattern, and walking behaviours using a custom algorithm based on a previously established gait model [46,47,72].

### 4.5. Future Considerations

For future clinical applications, longitudinal observational studies are required in order to measure inter- and intra-subject variability. However, such studies may be difficult to conduct because the deployment of the wearables at a large scale can be financially and technically challenging. For example, such studies may require a system with an unlimited Bluetooth connection to transmit the data, which can result in data loss. Consequently, it is important to establish a new way of sharing a platform where data will be stored securely [73]. There are many debates around using commercial devices versus research-grade devices in clinical trials. Due to disparities between data processing software and algorithms, it is difficult to say which method provides the best path for analysing wearable sensor data [74]. While it is encouraging that PD-specific algorithms have been validated [19,29,38,47], further investigation in this area is essential to propose the optimal algorithm that can identify specific clinical features to differentiate various NDDs.

Another research direction is to improve the devices used to collect data. An empirical comparison of devices is needed to find out the divergence between products and the efficacy, effectiveness, and ecological validity of wearable systems could be improved. Another possible area of future research could address the development of wearable sensing technology which is better adapted to the patient [75]. For example, such devices will need to be miniaturised, cost-reliable, and provide flexibility to the end-users (for example using nanotechnology to make material more adapted to the patient) [76]. It is also vital to take individual differences in acceptability and comfort into account when designing technologies for the NDD population. A compatible software network system should also be introduced which is intuitive and user-friendly to promote e-Health. It could improve routine care for patients, improve the physician-patient relationship, and increase autonomy [73]. Therefore, it may be interesting to introduce a novel remote monitoring technique to revolutionise healthcare management.

We also recognise that there are limitations to this review We pragmatically chose to focus on common NNDs and acknowledge that measures based on accelerometry have been tried in other patient populations, including patients with HD. Our criteria were such that some papers were excluded, as PA outcomes were not explicitly reported. Given the paucity of published studies on using wearable GPS, it is unlikely that limiting our search strategy to the last 5 years resulted in any GPS publications being omitted. However, as accelerometers have been widely used in health research for longer, we may have excluded older studies which used these devices in NDDs.

## 5. Conclusions

We sought to determine the extent to which wearable technologies that incorporate GPS and/or accelerometer technologies are being used in patients with NDDs. A systematic search identified only one study, in which GPS technology was used to measure mobility in PD, that performed well against the current gold standard interview-based techniques. In contrast, we found that a variety of devices containing accelerometers have been successfully applied to patient populations, including in well-organised clinical trials. Objective accelerometer-derived measures typically correlated with standard measures of PA and activities of daily living (ADL). This is a particularly promising development for a population of patients who frequently struggle to self-complete standard questionnaire-based tools, administered on an intermittent basis. Furthermore, a number of studies reported that these metrics correlated with disease progression or that they were capable of discriminating between disease stages.

The COVID-19 pandemic has been a catalyst for accelerating the use of digital healthcare solutions in clinical and research practice. To what extent these technologies mitigate or indeed further augment the isolation that older people experience as a result of the “technology gap” [77] remains to be determined. This review has demonstrated areas of success and potential, as well as highlighted areas that will benefit from a stronger evidence base with further development of the technologies and algorithms. Accessible, unobtrusive, acceptable monitoring that operates in a “closed-loop” system of feedback and intervention is a new frontier in neurodegenerative medicine and ageing. The COVID-19 pandemic has necessitated the rapid deployment and uptake of technologies by healthcare professionals and patients. Maintaining this pace and embedding technology in research and clinical practice would be a much needed step in the testing of new therapies, monitoring of disease progression, and risk prediction in order to attenuate the burden and negative impact borne by those affected by neurodegenerative conditions.

## Figures and Tables

**Table 1:** Summary of identified papers where GPS and accelerometer devices have been used in patients with neurodegenerative disorders. Participant age is presented as mean (SD) where available.

Author (Year)	Study Population	Device; Anatomical Site	Duration	Study Design	Reported Sensor Derived Outcome Measures	Data Processing Details	Key Findings
		Wearable GPS based studies					
Zhu et al. (2020) [15]	PD, *n* = 54, age 67.5 (6.3) yr	WIMU-GPS; upper back	14 days (waking hours)	Cross-sectional	Trip frequency, duration outside, life space size	Displacement and LSM data reported using a geospatial statistical approach based on computation of minimum span ellipse fitting all data points for each individual. Surface area of minimum span ellipse used to quantify life space size. Excluded if <6 days (>10 h/d) wear.	Reasonable agreement between GPS data, LSA, and mobility diaries for trip frequency and duration, but not life space size. GPS may help overcome the floor and ceiling effects of the LSA.
		Accelerometer based studies					
van Uem et al. (2018) [26]	PD, *n* = 39; PDD *n* = 8, age 69 yr (mean of both groups)	DynaPort Minimod; lower back	3 days	Cross-sectional	Time spent in sedentary and active episodes, TEE, number of ”bouts”, mean bout length	Data collected at 100 Hz. Self-report of non-wear and special activities (i.e., sports). Raw data analysed with manufacturer’s proprietary algorithms.	Total energy expenditure predicts PDQ-SI and PDQ-Mobility scores. Sedentary activity predicts PDQ-Mobility.
Pradhan & Kelly (2019) [27]	PD, *n* = 30, age 68.6 (5.9) y; healthy older adults *n* = 30, age 67.4 (4.7) yr	Fitbit Charge HR; wrist	14 days	Cross-sectional	Step count, number of bouts, timing of bouts of activity	PA intensity determined by proprietary algorithms that define MET values based on steps/minute and heart rate activity. PA intensity defined as: very active (≥6 METs); fairly active (3–6 METs), lightly active (1–3 METs), and sedentary.	Individuals with PD spent fewer minutes per day in moderate/vigorous activity, more minutes sedentary vs. healthy controls.
Mantri et al. (2019) [28]	PD, *n* = 30 #, median (IQR) age 70 (69–74) yr	Actigraph GT3X; waist	7 days	Cross-sectional	Step count, MVPA	Raw activity data was analysed in 30 s epochs. PD-specific algorithm [29] used to define steps and activity intensity. MVPA defined as >3.0 METs, or the equivalent of >1.31 m/s brisk walk. Valid wear time ≥ 10 h, excessively high counts excluded.	Median step count correlates with PASE.
Sulzer et al. (2021) [30]	PD, *n* = 45 #, median (range) age 71 (44–80) yr	DynaPort Minimod; lower back	3 days, data only collected at baseline	Longitudinal	Time spent in physical behaviour, number of behaviour bouts, timing of bouts of activity, step count, TEE	Manufacturer’s proprietary algorithms used to define posture/activity. Lying and sitting combined as SB. Standing, shuffling, and walking periods combined as active behaviour. For valid days (24 h registration, wear time > 80%), behaviours further categorised by time spent in behaviour, no. of behaviour bouts (i.e., >1 s, walking > 3 consecutive steps), mean bout length. TEE and step count calculated.	Longer sedentary bouts predict illness and death.
Moyle et al. (2017) [24]	Dementia, *n* = 192, age 85.5 (7.7) yr	SenseWear activity-armbands; arm	24 h	Cross-sectional	Step count, TEE, MET, Time spent physically active, lying down, awake, asleep	All data recorded in 60 s epochs. Analysed with manufacturer’s proprietary algorithms based on AI. Valid wear-time defined as ≥21 h.	Individuals in care home engaged in light physical activity, varying dependent on mobility factors, age, and sex.
Varma & Watts (2017) [31]	Alzheimer’s disease, *n* = 39, age 73.5 (7.9) yr; controls, *n* = 53, age 73.2 (6.5) yr	Actigraph GT3X+; hip	7 days	Cross-sectional	Intensity, activity type	Aggregation of VM data into 60 s epochs, average VM calculated for minute. Excluded days with <10 h of wear time. Wear/non-wear time classification algorithm used [32]. Intensity defined as 0–149 CPM for SB, 150–2689 CPM for light-intensity, and ≥2690 CPM for MVPA.	Mild AD is associated with lower intensity and complexity of the activity. Not associated with longer sedentary activity.
Kim et al. (2019) [20]	PD, *n* = 46, age 68 (7.9) yr	ActiGraph GT3X+; wrist and waist	7 days	Cross-sectional	Step count, activity count, time spent in activity type	Activity levels defined as SB (≤100 CPM), light (101–1951 CPM), and moderate (1952–5737 CPM) to vigorous PA (≥5738 CPM). PA intensity determined using cut-off points [33]. Walking speed classed as <1.04 m/s, 1.05 to 1.3 m/s, and > 1.31 m/s. PD-specific algorithm applied to percent time spent in different walking speeds [34].	Higher numbers of steps recorded with wrist accelerometer versus waist accelerometer, however, remained proportionate. Variation in recordings linked to increased tremor and bradykinesia.
Klenk et al. (2016) [35]	NDD (*n* = 103; *n* = 34 ataxia, age 58(11.3) yr, *n* = 15 PSP, age 66.2 (5.5) yr, *n* = 16 PD, age 72.2 (6.5) yr); *n* = 18 healthy young adults, age 28.3 (9.6) yr; *n* = 38 healthy older adults age 70.6 (3.9) yr	ActivPAL3; thigh	7 days	Cross-sectional	Walking duration, step count, cadence, walking bout length, absolute and relative number of walking bouts, number of daily sit-to-stand transfers	Manufacturer’s proprietary algorithms detect upright posture and classified activities as lying or sitting, standing, and walking. Days < 24 h data considered invalid.	Daily walking duration decreased for PD, ataxia and PSP groups versus health controls. Daily walking duration was lower for PD, ataxia and PSP groups compared to healthy controls; walking duration diminished as the diseases progressed.
Porta et al. (2018) [36]	PD, *n* = 18, age 68 (10.8) yr	ActiGraph GT3X; wrist	3 months	Cross-sectional	Step count, intensity of activity by time period	Data collected using 60 s epochs at 30 Hz, processed in manufacturer’s software. Valid wear-time >16 h/day. Step counts and PA classification based on the cut-off points for wrist-worn accelerometry in older adults [37], and (hip-worn) PD specific algorithms [29,38]. Nero algorithm provides walking speeds cut-points.	Peaks of physical activity in early morning and early evening. Physical activity intensity correlates with quantitative spatiotemporal and kinematic gait parameters.
van Eijk et al. (2019) [39]	Amyotrophic lateral sclerosis, *n* = 42, age 60 (12) yr	ActiGraph GT9X Link; hip	7 days every 2–3 months	Longitudinal	Activity count, MET	Data captured at 30 Hz, processed using manufacturer’s software in 10 s epochs using low-frequency extension (LFE) algorithm. Non-wear time algorithm applied [32], no activity defined as 150 min in advanced disease. Days < 8 h wear-time excluded. Percentage of time active estimated as the proportion of VM counts > 100 CPM. VM counts translated to METs to summarize average daily MET score.	Decline in physical activity of 0.64% per month. Correlation in decline of activity with ALSFRS daily function score.
Cerff et al. (2017) [40]	PD, *n* = 48: *n* = 17 PD-NC age 71 yr, *n* = 22 PD-MCI age 68 yr, *n* = 9 PDD age 72 yr	DynaPort Minimod; lower back	3 days	Cross-sectional	Step count, posture, Type, duration, intensity and volume of activity MET	Data collected at 100 Hz and a resolution of 1 milli *g* -force, manufacturer’s algorithms defined posture/activities. Lying and sitting combined as SB. Standing, shuffling, and walking combined as active behaviour. Days with <24 h, or wear time <80% excluded.	Increased number and longer sedentary bouts for individuals with PD dementia versus PD MCI and PD with cognitive impairment.
Paul et al. (2016) [41]	PD, *n* = 92, median (IQR) age 67.3 (7.8) yr	StepWatch 3 Step Activity Monitor; ankle	7 days	Cross-sectional	Step count, intensity	Stride counts recorded in 1-min intervals, manufacturer’s software used to transform stride counts into step counts (i.e., step count = stride count × 2) and to calculate amount of step activity, and minutes/day of MVPA (i.e., minutes > 100 steps/minute).	Two days of monitoring determined as adequate period to obtain sufficient activity level estimates.
Elazari et al. (2016) [42]	PD, *n* = 99 age 64.8 (9.5) yr; healthy older adults, *n* = 38 age 78.7 (4.4) yr	Dynaport Hybrid; lower back	3 days	Cross-sectional	Type, duration, posture	Data recorded at 100 Hz, analysed using MATLAB. Activities detected automatically based on the local mean of acceleration signals. Transitions between activity segments automatically determined. ML algorithm applied to assess the ability of the entire feature set to discriminate between PD and non-PDs. The algorithm identified the features of PD using SVM in MATLAB.	Machine learning algorithm is able to differentiate walk-to-sit and sit-to-walk transitions which vary according to the severity of PD and between patients with PD and healthy older adults.
Nero et al. (2016) [34]	PD, *n* = 91, age 73 (6) yr	Actigraph GT3X; hip	7 days	Cross-sectional	Duration, type	Manufacturer’s software used to process raw data in 15 s epochs, episodes of ≥90 min of consecutive 0 s considered non-wear and excluded. Data excluded <4 days data. Normal data band-pass filter option utilized. PA expressed per day in total activity counts (TAC). Brisk walking (>1.0 m/s) in minutes/day calculated [43]. Sedentary time calculated using cut-off points [44].	Motor impairment is negatively associated with PA. Reported an association between PA and both physical function and balance. Motor impairment, body mass index and dyskinesia contribute to the variation in PA levels. PA and brisk walking had principally different associated factors.
Loprinzi et al. (2018) [45]	PD, *n* = 25, 68.7 yr	ActiGraph GT1M; hip	1–2 weeks	Cross-sectional	Type, intensity	Minimum of 4 days with ≥10 h per day data were included in the analyses. Non-wear defined by a period of a minimum of 60 consecutive minutes of zero activity counts, with the allowance of 1–2 min of activity counts between 0 and 100. Activity CPM of ≥1952 denoted MVPA intensity.	Regardless of motor impairment, age and gender. MVPA improves cognition quantified with MoCA.
Buckley et al. (2020) [46]	Aged residential care residents, *n* = 257, age 84.5 (7.2) yr, Dementia, *n* = 34, age 81.53 (7.07) yr	Axivity AX3; lower back	7 days	Cross-sectional	Steps, volume, walking time, bouts per day, pattern or variability of activity behaviours	Programmed to sample acceleration at a frequency of 100 Hz (range ±8 g). Only participants who had at least 3 full days of analysis. Custom automated MATLAB algorithm calculated PA walking outcomes according to a previously established gait model [19,47]	Optimal number of days recording for reliable estimate of activity determined to be between 2–5 days, dependent on required level of dementia care.
van Alphen et al. (2016) [48]	Institutionalised dementia, *n* = 83, age 83 (7.6) yr Community dwelling dementia, *n* = 37, age 77.3 (5.6) yr Healthy older adults, *n* = 26, age 79.5 (5.6) yr	Actiwatch AW-4; wrist	6 days	Cross-sectional data from a longitudinal study	Sedentary behaviour, type, Intensity, amount, duration, patterns	Data collected at 32 Hz using 60 s epochs. Raw data, evaluated without cut-off points. 0 assigned to epochs without acceleration. PA quantified as time spent in specific zones of activity counts with ranges of 100 CPM, <100 CPM classed as SB.	Institutionalised individuals with dementia are more sedentary than community-dwelling individuals with dementia who in turn are sedentary for longer than healthy controls.
von Rosen et al. (2021) [49]	PD, *n* = 301, age 71.4 (6.4) yr	Actigraph GT3X+; hip	7 days	Cross-sectional	Duration, type, intensity, sedentary behaviour	Manufacturer’s software used to process raw data, collected using 60 s epochs. Non-wear defined as ≥60 min of 0 counts, allowing for 2 min of counts between 0 and 100. Data included from participants with at least 1 day with ≥10 h wear time. Epochs classified as intensity levels using cut-off points for older adults: SB (<100 CPM), LIPA (100–1040 CPM), and MVPA (≥1041 CPM) [50].	The group categorised as being ”sedentary” as opposed to “light-mover” or “steady-mover” performed worse on tests of balance, mobility, and had an increased likelihood of falling.
Moyle et al. (2018) [51]	Dementia, *n* = 455, age 85.3 (1.0) yr	SenseWear Professional 8 armband; arm	24 h at baseline, during two single days in weeks 5, 10 and 15	Longitudinal/RCT	Step count, duration, MET Time spent lying down, awake, and asleep	Manufacturer’s proprietary software and algorithms used. Data recorded in 60 s epochs. Valid wear time for analysis defined as ≥21 h.	A robotic seal intervention given to individuals cared for in a dementia care home led to a reduction in step count both during the daytime and at night and a reduction in daytime PA.
Murphy et al. (2017) [52]	Dementia, *n* = 20, age 78.7 (11.8) yr	Sensewear Armband; arm	7 days	Cross-sectional	TEE, step count, sedentary behaviour, duration	Limited data processing information provided.	TEE positively correlated with weight and negatively correlated with duration of sleep.
Leavy et al. (2021) [53]	PD, *n* = 89, age 71.0 (6.0) yr	Actigraph GT3x; hip	7 days	Cross-sectional	Intensity, step count, duration, sedentary behaviour	Manufacturer’s software used to process raw data, collected in 60 s epochs. Non-wear defined as ≥60 min of 0 counts, allowing for 2 min of counts between 0 and 100. Data included if ≥4 days with ≥9 h wear-time. Epochs classified as intensity levels using cut-off points: SB (<100 CPM), LIPA (100–1040 CPM), and MVPA (≥1041 CPM) [54,55].	Data collected during COVID-19 pandemic. 67% reported a pandemic-related reduction in exercise habits. Being female, aged over 70 years and older, and mobility problems associated with being less physically active.
Kok et al. (2017) [56]	Dementia, regular special care unit *n* = 48, age 82.9 (8.3) yr Small-scale homelike SCU, *n* = 67, age 83.3 (6.3) yr	Actiwatch; wrist	7 days	Longitudinal study, data collected at 3-month intervals	Duration, sleep activity	Limited data processing information provided. Incomplete recordings were excluded from analysis. Valid data was collected 7 × 24 h for each measurement. In case missing or invalid data device worn for one more week.	Patients with dementia moved from regular Special Care Unit (SCU) to a small scaled homelike SCU. No effect on activity levels between groups following intervention.
Hartman et al. (2018) [57]	Dementia *n* = 45, age 79.6 (5.9) yr Control, *n* = 49, age 80.0 (7.7) yr	Phillips Acti-watch 2; wrist	7 days	Cross-sectional	Sedentary behaviour, intensity, duration	Manufacturer’s software used to export data, sleep intervals manually set and excluded by custom software in MATLAB. Measures obtained every 30 s, wrist accelerations translated to counts to estimate PA/SB. Data included if ≥6 valid days (>10 h). Counts per epoch converted to CPM. Cut-off points of 145, 145–274, 274–597, and >597 CPM used for SB and very light, light-to-moderate, and MVPA. Prolonged SB defined as 30 min with <145 CPM without 1 min of >145 CPM.	People with dementia demonstrated increased sedentary periods and decreased time in light-moderate and moderate-vigorous activity as compared with healthy controls.
Fleiner et al. (2016) [58]	Dementia, *n* = 45, age 45 (7) yr	uSense sensor device; lower back	3 days	Cross-sectional as part of an RCT	Posture, intensity, type, sedentary behaviour, duration	Data sampled at 100 Hz, limited data processing information provided.	Overall low level of activity and wide variety of activity patterns observed. Patients spent 45% of their time lying, 41% sedentary while sitting or standing, 7% active while sitting, and 7% walking.
Garshol et al. (2020) [43]	Dementia, farm-based *n* = 29, age 74.0 (7.2) yr Regular day care, *n* = 39, age 83.4 (8.1) yr	Actisleep+ and Actigraph; wrist	7 days	Cross-sectional	Intensity, sedentary activity, duration, step count	Manufacturer’s software used to report PA, limited data processing information provided.	Higher levels of moderate activity were observed in individuals attending farm-based day care activities versus standard care, whilst similar time spent on sedentary or light activity for both groups.
Adams et al. (2017) [59]	PD, *n* = 16, age 68 (8.7) yr HD (*n* = 15), age 55.0 (10.7) yr Prodromal HD, *n* = 5, age 38 (8.6) yr Controls without a movement disorder *n* = 20, age 58 (16.2) yr	BioStampRC; placed on chest, limb, thigh and forearm	2 days	Cross-sectional	Posture, duration, type	Data collected at 31.25 Hz. Chest and thigh sensors identified posture. Further analysis differentiated walking and standing. Walking durations identified with normalized autocorrelation-based analysis [60]. Walking split into epochs within upright posture intervals, lags of 0.3–1.2 s considered as plausible range of step durations.	Individuals with PD spend less time lying down than those with Huntington’s disease but a similar amount of time to healthy controls.
Feng et al. (2020) [61]	Case control study: Idiopathic REM sleep behaviour disorder (iRBD), *n* = 88, age 69.8 (7.7) yr Non-RBD controls, *n* = 44, age 70.1 (10) yr Clinically diagnosed alpha-synucleinopathies *, *n* = 44, age 70.7 (8.8) yr Prospective nested case-control study: non-convertors **, *n* = 66, age 70.9 (7.5) yr; convertors, *n* = 22, age 72.1 (7.6) yr	Actiwatch Spectrum Plus, wrist	7 days	Case-control study and a prospective nested case-control study	Pattern, duration, sleep-activity, type (rest-activity patterns)	Manufacturer’s software used to report data by 60 s epochs. Data excluded as invalid/missing data if exceeded 33% of total for day/interval.	Actigraphy-derived napping (percentage of time, episodes, and duration) occurred more in those with PD and DLB, than iRBD and, in turn, controls with a similar pattern of decreasing activity across the three groups. More napping and lower activity levels were seen in the iRBD group who had a clinically diagnosed alpha-synucleinopathies (at ~2 years) as compared with those that had not “converted”.

*n*, number; PD, Parkinson’s disease; AD, Alzheimer’s disease; ALS, amyotrophic lateral sclerosis; DLB, dementia with Lewy bodies; IMU, inertial measurement unit; iRBD, idiopathic rapid eye movement sleep behaviour disorder; GPS, global positioning system; PA, physical activity; LSA, life space assessment; PDQ, Parkinson’s Disease Questionnaire; TEE, total energy expenditure; ALSFRS-R, the ALS Functional Rating Scale; VM, vector magnitude; MVPA, moderate-to-vigorous physical activity; PASE, Physical Activity Scale for the Elderly; PSP, progressive supranuclear palsy; MET, metabolic equivalent of task estimates; MoCA, Montreal Cognitive Assessment; MCI, mild cognitive impairment; REM, rapid eye movement; SB, sedentary behaviour; CPM, counts per minute; SVM, support vector machine; AI, artificial intelligence; IQR, interquartile range; * alpha-synucleinopathies are the specific spectrum of neurodegenerative conditions that include PD; ** converters are individuals with REM sleep behaviour disorder who go on to develop PD, a known risk factor [62]; #, sensor data from subset.

## Data Availability

Not appliable.

## Supplementary Materials

The following are available online at https://www.mdpi.com/article/10.3390/s21248261/s1, Table S1: Search strategy table (SST) detailing search terms entered in COVID for four electronic databases (MEDLINE, EMBASE, AMED and APA PsycInfo).
