# Supplementary material for: Wearable GPS and Accelerometer Technologies for Monitoring Mobility and Physical Activity in Neurodegenerative Disorders: A Systematic Review
Source: Sensors (Basel). 2021 Dec 10;21(24):8261. doi: 10.3390/s21248261 (PMC8705556; doi:10.3390/s21248261)
Supplement: Supplementary file 1 [file sensors-21-08261-s001.zip › sensors-1429776-supplementary.pdf]

Supplementary material

Table S1. Search strategy table (SST) detailing search terms entered in OVID for four electronic databases (MEDLINE, EMBASE, AMED and APA PsycInfo).

#	Searches	Results
1	exp Neurodegenerative Disease/	1046925
2	(neuro* adj3 degenera*).mp.	51670
3	(neurodegenerative or neurological* or nervous system).mp	2444900
4	exp Parkinson disease/	240245
5	(Parkinson or parkinson*).mp	404634
6	Parkinson Disease/ or Parkinsonian Syndrome/ or Parkinsonian Disorders/ or Parkinsonian/ or Parkinsonism/ or Parkinsonian/ or Parkinsonism/	270674
7	Motor Neuron Disease/ or Amyotrophic Lateral Sclerosis/	77486
8	exp Alzheimer Disease/	367517
9	Alzheimer/ or Alzheimers.mp.	409100
10	Vascular Parkinsonism.mp	1191
11	exp Dementia/	652340
12	Vascular dementia.mp. or exp Dementia, Vascular/	30175
13	(movement or motor activity or mobility or monitoring ambulatory or physical activity).mp.	1981615
14	exp Geographic Information System/	20034
15	(GPS or gps or geographic Information System or differential Global Positioning system or global navigation satellite system or GLONASS or Geographic navigation satellite system or satellite communication system).mp.	89008
16	14 or 15	94698
17	exp Accelerometry/	18901
18	(Accelerometer or Accelerometry or Inertial measurement unit).mp.	49598
19	17 or 18	52897

20	(wearable electronic devices or wearable sensors or wearables or wearable*).mp.	39766
21	1 or 2 or 3	3279845
22	4 or 5 or 6 or 7	471780
23	8 or 9 or 10 or 11 or 12	759095
24	21 or 22 or 23	3625106
25	16 or 19 or 20	180704
26	24 and 25	9871
27	limit 26 to english language	9527
28	limit 27 to human [Limit not valid in AMED; records were retained]	8470
29	limit 28 to humans [Limit not valid in AMED,APA PsycInfo; records were retained]	8470
30	limit 29 to last 5 years	4972

Database : Ovid Medline (R) shows the total number of papers with each search terms from August 2021 to September 2021.
